# Comparative Efficacy of Integrated Stent-Graft versus Conventional Covered Stent-Graft Alone in Type B Aortic Dissection: A Retrospective Study Focusing on Aortic Remodeling and Branch Perfusion

**DOI:** 10.31083/RCM49740

**Published:** 2026-06-24

**Authors:** Jieyu Cao, Zhengwen Lei, Keke Xu, Shuangxi He, Jun Hu, Linfei Xing, Juan Luo

**Affiliations:** ^1^Department of Cardiothoracic Surgery, The First Affiliated Hospital, Hengyang Medical School, University of South China, 421001 Hengyang, Hunan, China; ^2^School of Nursing, University of South China, 421001 Hengyang, Hunan, China; ^3^Department of Cardiothoracic Surgery, The Second Affiliated Hospital, Hengyang Medical School, University of South China, 421001 Hengyang, Hunan, China; ^4^Hengyang Medical School, University of South China, 421001 Hengyang, Hunan, China

**Keywords:** type B aortic dissection, thoracic endovascular aortic repair, integrated stent-graft system, bare-metal stent, aortic remodeling, branch artery

## Abstract

**Background:**

To compare the early efficacy of an integrated stent-graft (16-cm covered stent combined with a 6-cm distal bare stent) versus a conventional covered stent-graft alone in patients with acute type B aortic dissection (TBAD), with a focus on aortic remodeling and branch artery perfusion.

**Methods:**

In this single-center retrospective cohort study, 102 patients were included (integrated stent-graft: n = 34; conventional stent-graft: n = 68). Aortic remodeling was assessed using the true lumen area ratio at five predefined anatomical planes. Branch artery perfusion patterns and procedure-related complications were evaluated preoperatively and at 3-month follow-up.

**Results:**

At 3 months, the integrated stent-graft group demonstrated significantly higher true lumen area ratios at the left subclavian artery plane 0.60 (0.41, 0.63) vs. 0.41 (0.25, 0.54), *p* = 0.002; pulmonary artery plane 0.65 (0.54, 0.71) vs. 0.44 (0.38, 0.53), *p* < 0.001; and diaphragmatic plane 0.61 (0.50, 0.76) vs. 0.44 (0.37, 0.51), *p* < 0.001. No significant differences were observed at the renal or iliac planes. Regarding branch perfusion, a significant overall difference in postoperative perfusion pattern distribution was observed for the celiac trunk (*p* = 0.027) and left renal artery (*p* = 0.017). No differences were detected in other branch arteries. The incidence of postoperative new entry tears was lower in the integrated stent-graft group (23.5% vs. 41.2%), although this difference did not reach statistical significance (*p* = 0.079).

**Conclusions:**

In this exploratory retrospective study with short-term (3-month) follow-up, the integrated stent-graft system was associated with improved early true lumen remodeling in the proximal and mid-aortic segments and differences in branch artery perfusion patterns compared with a conventional covered stent-graft alone. A trend toward fewer new entry tears was observed, but given the limited follow-up and sample size, these findings should be considered preliminary. Confirmation in larger prospective studies with long-term follow-up is warranted to determine whether these early imaging changes translate into durable clinical benefit.

## 1. Introduction

Aortic dissection is a life-threatening vascular emergency characterized by abrupt onset and severe clinical manifestations. Type B aortic dissection (TBAD), which involves the descending aorta, accounts for approximately one-third of all cases [1]. The treatment and management strategies for this condition have garnered significant attention in recent years. Initial management typically entails rigorous blood pressure and heart rate control to reduce shear stress on the aortic wall, thereby preventing rupture and slowing disease progression. However, despite conservative treatment, approximately one-third of patients progress to complex conditions requiring surgical intervention [2]. If not managed appropriately, patients remain at serious risk of aortic rupture, end-organ ischemia, and failure, leading to poor long-term outcomes [3].

Thoracic Endovascular Aortic Repair (TEVAR) has emerged as an innovative treatment for complex TBAD. By sealing the primary proximal entry tear, restoring true lumen flow, and promoting false lumen thrombosis, TEVAR has become a first-line therapeutic option for this condition [4,5]. However, the standard TEVAR procedure, which involves covering the primary tear with a stent-graft, has certain limitations. Firstly, the rigid distal end of the stent-graft can create a focal stress concentration on the fragile aortic intima, potentially leading to postoperative distal stent graft-induced new entry (dSINE). This phenomenon is recognized as a major complication following TEVAR and can precipitate new dissections or aneurysmal dilatation [6]. Secondly, for dissections with extensive involvement, a proximal stent-graft alone is often insufficient to achieve satisfactory remodeling of the distal aorta (particularly the abdominal segment) and adequate reperfusion of branch arteries. Persistent false lumen perfusion predisposes to “aortic remodeling failure”, increasing the risks of aneurysm formation, rupture, and adversely impacting long-term patient survival [7,8].

To overcome the limitations of conventional TEVAR, a combined strategy involving the distal extension of the stent-graft with a bare-metal stent (e.g., the PETTICOAT technique) has been explored. This approach aims to utilize the radial force of the bare stent to stabilize the distal true lumen, maintain branch artery patency, and theoretically reduce the risk of new entry tears by distributing mechanical stress [9,10]. Studies have confirmed that this technique significantly promotes aortic remodeling, particularly true lumen expansion and false lumen regression in the abdominal aorta region, while also helping to stabilize the intimal flap and preserve branch artery flow [11,12]. Furthermore, this strategy has been shown to effectively reduce the incidence of stent graft-induced new entry (SINE) [13,14]. However, the traditional implantation of modular stent systems carries potential risks such as misalignment, endoleak at the junction, or device migration. In this context, single integrated stent-graft system (16-cm covered stent combined with a 6-cm distal bare stent) that combine the stent-graft and bare stent into one unit have been developed. These integrated systems hold promise for inheriting the advantages of the combined strategy while potentially offering superior mechanical integrity and procedural convenience.

Previous studies have confirmed that the strategy combining a covered stent with a distal bare stent (e.g., the PETTICOAT technique) offers potential advantages in promoting distal aortic remodeling for TBAD [12]. However, most of these studies were based on modular stent systems, in which the proximal covered stent and the distal bare stent are implanted sequentially. In contrast, the present study evaluates a novel integrated stent-graft system that incorporates a 16 cm covered stent and a distal 6 cm bare stent into a single integrated unit, designed to achieve smoother mechanical force transmission and better conformability. Currently, systematic comparative studies on this specific integrated stent design versus conventional covered stents alone in the treatment of TBAD remain limited. Therefore, this study aimed to compare the differences between the integrated stent and the conventional stent in the treatment of TBAD through a retrospective cohort analysis. The primary objectives were to compare: (1) aortic true lumen remodeling, assessed by the true lumen area ratio at five predefined anatomical planes at 3-month follow-up; and (2) branch artery perfusion patterns, classified as true lumen, false lumen, or mixed perfusion for six major branch arteries at 3-month follow-up. The secondary objectives were to compare: (1) the incidence of postoperative new entry tears; and (2) other procedure-related complications. We hypothesized that the integrated stent-graft system might be associated with improved true lumen remodeling, enhanced true lumen perfusion to critical branch arteries, and a lower incidence of new entry tears compared with conventional stent-graft alone.

## 2. Materials and Methods

### 2.1 Study Design

**Fig. 1.**
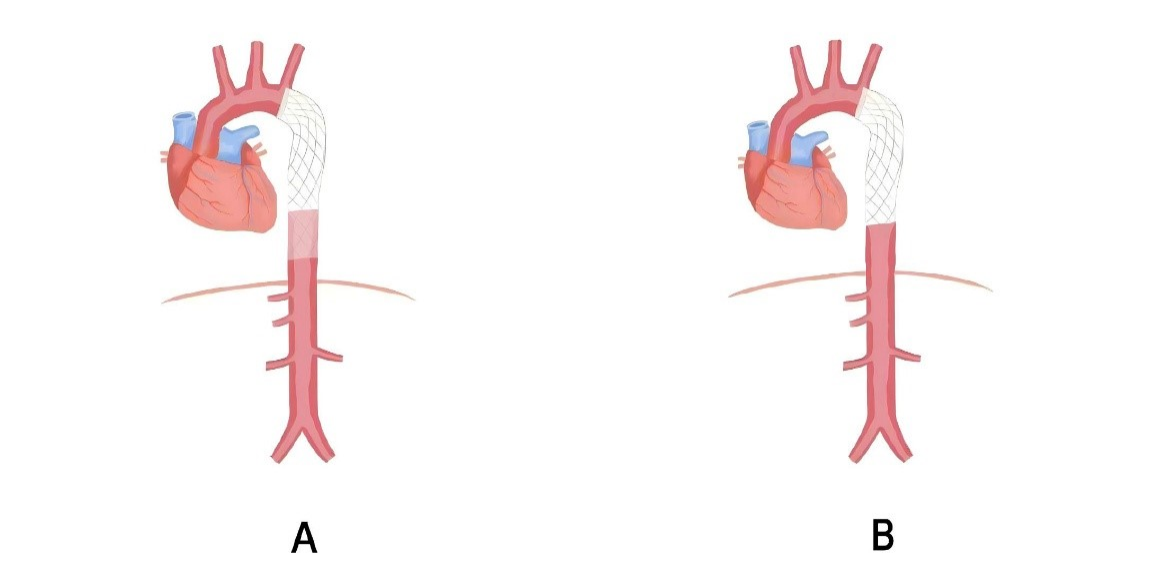
**Schematic diagrams of the two stent-graft structures**. (A) Experimental group: integrated stent-graft system. (B) Control group: conventional Stent-Graft.

This was a single-center retrospective cohort study. The experimental group received treatment with an integrated stent-graft system (16-cm covered stent combined with a 6-cm distal bare stent), while the control group received a 16 cm conventional Stent-Graft (structural schematic shown in Fig. 1). All patients underwent clinical and imaging assessments preoperatively and at three months postoperatively.

### 2.2 Ethical Statement

This study was approved by the Ethics Committee of The Second Affiliated Hospital, University of South China (Approval No. 2025050). Due to the retrospective design of the study, which involved no alteration to interventions, the requirement for informed consent from patients was waived by the ethics committee. All patient data were de-identified prior to analysis to protect patient privacy.

### 2.3 Study Setting and Period

This study was conducted at the Department of Vascular Surgery, The Second Affiliated Hospital, University of South China. Patients with TBAD admitted consecutively between January 2021 and February 2025 were included. Patients were identified and screened through the hospital’s electronic medical record system and picture archiving and communication system, encompassing all those who received TEVAR for TBAD.

### 2.4 Patient Identification

**Fig. 2.**
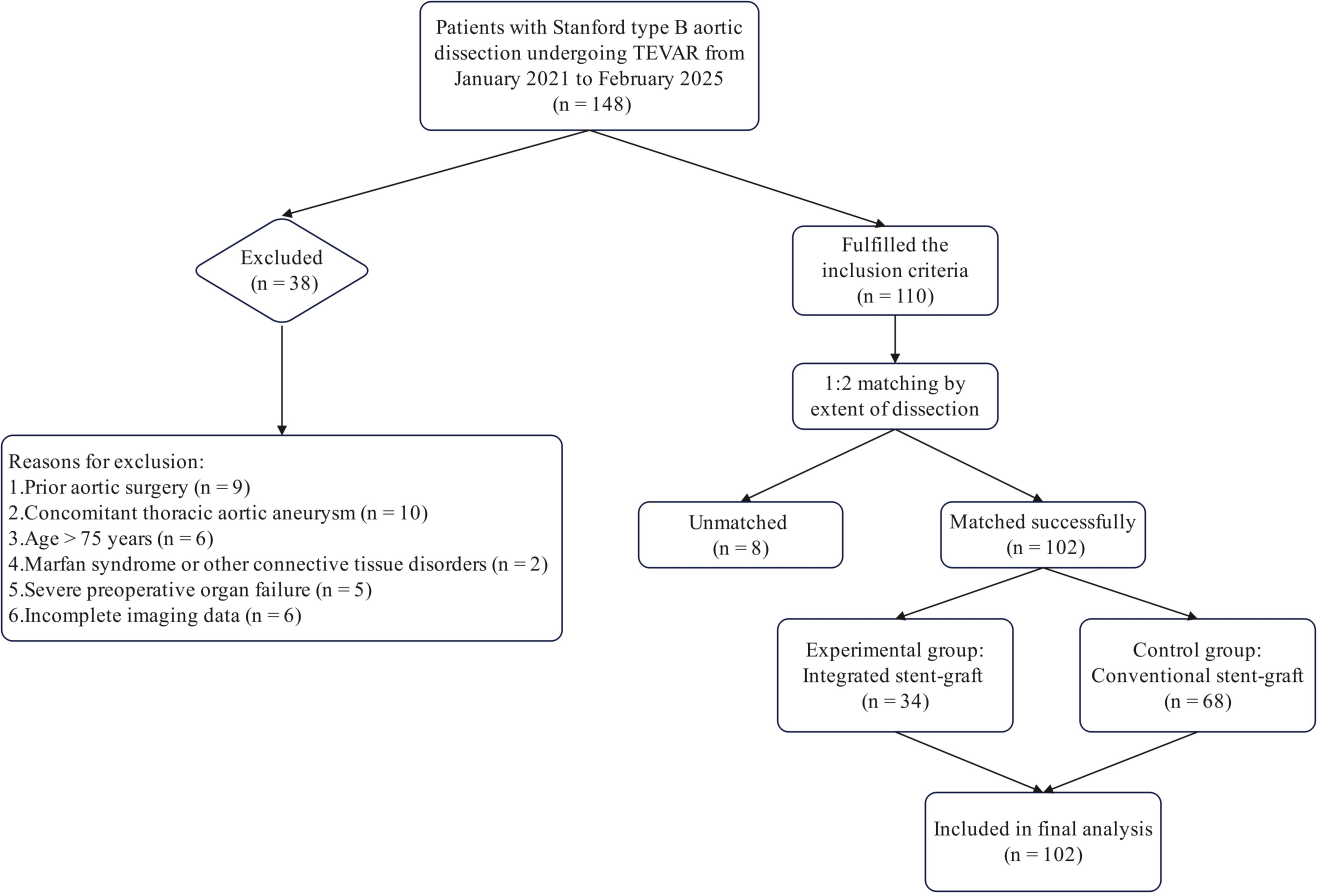
**Patient selection flowchart**. TEVAR, thoracic endovascular aortic repair.

Patients were identified through the hospital’s electronic medical record system and picture archiving and communication system (PACS). All consecutive patients who underwent TEVAR for TBAD between January 2021 and February 2025 were screened for eligibility. The screening process and reasons for exclusion are detailed in the patient flowchart (Fig. 2).

### 2.5 Inclusion and Exclusion Criteria

The inclusion criteria for this study were: (1) First-time diagnosis of acute TBAD, defined as a primary entry tear located in the descending thoracic aorta (distal to the left subclavian artery) with no involvement of the ascending aorta; retrograde extension into the aortic arch was permitted. “First-time diagnosis” was confirmed by reviewing medical records and previous imaging studies to ensure no prior history of aortic dissection (acute or chronic). Patients with recurrent acute TBAD (i.e., a new acute event in a patient with known chronic dissection) were excluded; (2) Undergoing first-time TEVAR; (3) Age between 18 and 75 years; (4) Preoperative computed tomography angiography (CTA) confirmed that the dissection did not involve the ascending aorta (i.e., Stanford type B). Patients with retrograde extension of the dissection into the aortic arch (e.g., from a zone 2 entry tear) were included as long as the ascending aorta was unaffected. All included patients had complicated TBAD, defined as the presence of at least one of the following: refractory hypertension despite optimal medical therapy, persistent or recurrent chest pain, rapid aortic expansion, malperfusion syndrome (visceral, renal, or lower limb), or contained rupture [15].

Exclusion criteria were: (1) Concomitant thoracic aortic aneurysm. This was defined as any segment of the thoracic aorta (ascending aorta, aortic arch, or descending thoracic aorta) with a diameter >50 mm or exceeding 1.5 times the expected normal diameter based on nomograms. A threshold of 50 mm was chosen to minimize confounding from pre-existing aneurysmal disease on aortic remodeling assessment, acknowledging that this is more conservative than the commonly used 55 mm threshold. This decision was made to enhance internal validity in this preliminary evaluation of a novel device; (2) Confirmed Marfan syndrome (according to Ghent criteria) or other hereditary connective tissue disorders; (3) History of prior aortic surgery (Prior aortic surgery was defined as any surgical procedure involving the thoracic or abdominal aorta, including aortic valve replacement, ascending aorta replacement, aortic arch repair, and previous TEVAR or open aortic surgery. Isolated coronary artery bypass grafting (CABG) that did not involve aortic manipulation (e.g., off-pump CABG with no aortic anastomosis) was not considered prior aortic surgery; however, no such cases were identified in the screened population. Patients with a history of CABG involving aortic anastomosis were excluded as this constitutes aortic manipulation). Patients with prior aortic surgery were excluded because previous surgical alterations could alter aortic wall structure and healing responses, potentially confounding the assessment of aortic remodeling and branch perfusion related to the current stent-graft; (4) Presence of severe preoperative organ failure (e.g., cardiogenic shock, renal failure requiring dialysis). Severe preoperative organ failure was considered a contraindication for TEVAR at our institution; therefore, such patients did not undergo the procedure and were consequently excluded from the study. This criterion thus reflects both clinical practice and study design.

### 2.6 Group Allocation

Patient grouping was based on the availability of the stent system at the time of the procedure and operator preference, representing non-random assignment. Patients who received the integrated stent-graft system were assigned to the experimental group, and those who received the conventional covered stent-graft alone were assigned to the control group.

### 2.7 Interventions

According to current guidelines [15], urgent TEVAR is indicated for patients with complicated acute TBAD to prevent life-threatening complications such as rupture or malperfusion. All procedures were performed by the same team of experienced vascular surgeons in a hybrid operating room during the acute phase (≤14 days from symptom onset). Preoperatively, all patients underwent CTA to assess dissection morphology. Stent size selection was based on the diameter of the proximal landing zone measured on preoperative CTA, with an oversize ratio controlled within the range of 0–10%. The experimental group received the integrated stent-graft system, while the control group received a 16 cm conventional covered stent-graft alone. Postoperatively, all patients received standardized antiplatelet therapy (aspirin 100 mg/day for at least 6 months) and underwent CTA follow-up at three months post-surgery [15].

The integrated stent-graft system used in this study was the Talos® straight thoracic aortic stent-graft system (MicroPort Endovascular, Shanghai, China). The device consists of a covered thoracic stent-graft combined with a distal bare stent extension designed specifically for the treatment of type B aortic dissection. The stent-graft is delivered through a dedicated endovascular delivery system via femoral arterial access under fluoroscopic guidance. The design incorporates an extended distal bare stent segment to improve distal true lumen expansion while maintaining perfusion of major branch vessels. The Talos® system has received regulatory approval under the European Union Medical Device Regulation (MDR 2017/745). Compared with conventional thoracic stent-grafts, the device is designed to provide enhanced radial support at the distal aorta while minimizing excessive radial force at the proximal landing zone, thereby potentially reducing the risk of distal stent-induced new entry tears.

### 2.8 Data Sources and Measurements

**Fig. 3.**
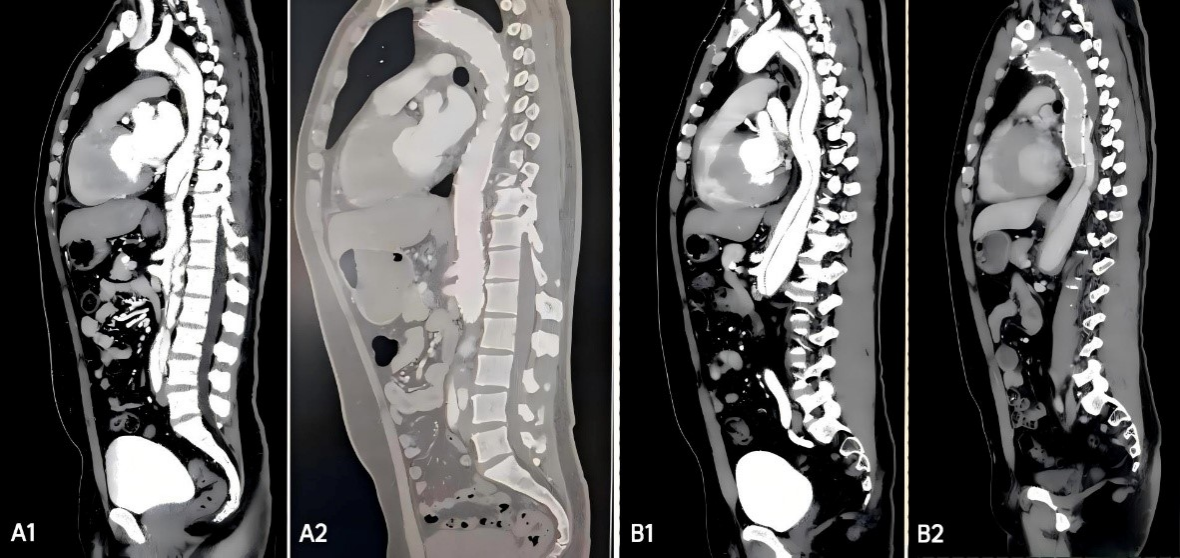
**Representative aortic CTA images illustrating remodeling**. (A1,A2): Preoperative and 3-month postoperative comparisons from a patient in the experimental group. (B1,B2): Preoperative and 3-month postoperative comparisons from a patient in the control group.

All patients underwent CTA examinations preoperatively and at three months postoperatively (representative images are shown in Fig. 3). All CTA acquisitions were performed using a standardized protocol with slice thickness ≤1 mm and isotropic voxel reconstruction. Images were acquired after intravenous injection of non-ionic contrast medium (350–370 mgI/mL) at a rate of 4–5 mL/s, with bolus tracking in the descending aorta. Image analysis was performed using a three-dimensional post-processing workstation (Syngo.via, Siemens Healthineers, or equivalent software) with the centerline multi-planar reconstruction method to ensure standardization and reproducibility. All imaging measurements were conducted collaboratively by two experienced radiologists who were blinded to group assignments; they independently evaluated the images, and any disagreements were resolved through consensus, with the final agreed-upon values recorded.

**Anatomical definitions and baseline variables**. The location of the primary entry tear was defined according to aortic anatomical zones (Zone 2: distal to the left subclavian artery; Zone 3: proximal to mid-thoracic aorta; Zone 4: distal thoracic aorta, etc.). The extent of dissection was categorized based on the most distal aortic segment involved by the false lumen: (1) thoracic aorta only (dissection confined to the descending thoracic aorta, corresponding to DeBakey type IIIa); (2) extending below the diaphragm into the abdominal aorta (DeBakey type IIIb). The five anatomical planes used for aortic remodeling assessment were defined as: (1) left subclavian artery plane (cross-section through the inferior margin of the left subclavian artery ostium); (2) pulmonary artery plane (cross-section at the level of the main pulmonary artery bifurcation); (3) diaphragm plane (cross-section at the level of the esophageal hiatus); (4) renal artery plane (cross-section through the superior margin of the left renal artery ostium); (5) iliac artery plane (cross-section 1 cm above the aortic bifurcation). Baseline variables collected included sex, age, time from symptom onset (hours), smoking history, blood pressure (systolic/diastolic, mmHg), comorbidities (coronary artery disease, diabetes mellitus, chronic obstructive pulmonary disease, etc.), cardiac function indices (left ventricular ejection fraction, %; left ventricular end-diastolic diameter, mm), and aortic morphological parameters (ascending aorta diameter, mm; aortic sinus diameter, mm).

**Primary measures of outcome. **The primary efficacy outcomes were true lumen area ratio and branch artery perfusion pattern, both assessed at the 3-month follow-up. (1) True lumen area ratio was defined as the ratio of the true lumen area to the total aortic lumen area at each of the five predefined anatomical planes. Identification of true and false lumens on CTA was based on the position of the intimal flap, contrast agent filling sequence, and thrombus morphology. Measurements were performed using centerline multi-planar reconstruction to ensure a perpendicular cut to the aortic long axis; the resulting ratio is dimensionless. (2) Branch artery perfusion pattern was evaluated for six major branch arteries: celiac trunk, superior mesenteric artery, left and right renal arteries, and left and right iliac arteries. Perfusion was classified as true lumen perfusion (the branch artery is supplied entirely by the true lumen, with no communication with the false lumen), false lumen perfusion (the branch artery is supplied entirely by the false lumen), or mixed perfusion (the branch artery receives blood supply from both the true and false lumens, either simultaneously or via an intimal tear connecting both channels). Perfusion success was defined as the branch artery being supplied entirely by the true lumen at the 3-month follow-up.

**Safety outcomes**. Safety outcomes were complications occurring within 30 days postoperatively and during the 3-month follow-up period. These included: (1) new entry tears — defined as new intimal tears located at the proximal or distal edge of the stent on postoperative CTA that were not present on preoperative imaging. To distinguish stent-induced new entry from natural disease progression, the following criteria were applied: (a) the tear must be immediately adjacent to the stent edge (within 5 mm); (b) no evidence of the tear on preoperative CTA; and (c) consensus agreement by two independent radiologists. Cases where the tear was clearly remote from the stent or associated with progressive dissection elsewhere were not classified as new entry tears related to the stent; (2) intestinal ischemia – diagnosed based on clinical manifestations combined with imaging or surgical confirmation; (3) hepatic insufficiency – new onset postoperative elevation of transaminases >3 times the upper limit of normal; (4) renal insufficiency – new onset postoperative increase in serum creatinine >50% or requirement for dialysis; (5) lower limb ischemia – diagnosed based on clinical manifestations confirmed by imaging.

### 2.9 Bias Control

To minimize selection bias and information bias, the following measures were implemented: (1) All surgeries were performed by the same team, and the follow-up protocol was standardized. (2) The matching process was carried out by a researcher blinded to the study outcomes. (3) Imaging data were evaluated jointly by two radiologists blinded to group allocation, with discrepancies resolved through consensus. (4) Statistical analyses were performed by a statistician who was not involved in data collection.

### 2.10 Sample Size Justification

No pre-study sample size calculation was performed for this retrospective study. As the integrated stent-graft system is a novel device introduced into clinical practice, there were insufficient preliminary data to reliably estimate expected effect sizes for a formal power analysis. Therefore, a consecutive enrollment strategy was adopted, including all eligible patients treated at our center during the study period (January 2021 to February 2025). The limitations related to the sample size, including the potential for type II error, are addressed in the Discussion section (4.4).

### 2.11 Statistical Analysis

To control for confounding bias and enhance intergroup comparability, a matching design was employed. The extent of dissection involvement (DeBakey type IIIa vs. IIIb) served as the primary matching variable. Patients in the experimental group were matched with patients in the control group at a 1:2 ratio by a researcher blinded to the study outcomes. Following matching on the primary variable, further tests were conducted to ensure the balance of key baseline covariates such as age, hypertension, and diabetes mellitus between the groups. The success of matching was confirmed by the absence of significant differences in baseline characteristics between the two groups (all *p* > 0.05) (see Table 1).

**Table 1.** **Baseline characteristics of patients**.

Variable		Experimental group (n = 34)	Control group (n = 68)	*p*-value
Male sex		24 (70.6)	52 (76.5)	0.520
Age		55 (52, 62)	56.5 (53, 68)	0.180
Time from onset (h)		7 (6, 48)	8.5 (5, 16)	0.991
Smoking history		20 (58.8)	44 (64.7)	0.562
Systolic blood pressure (mmHg)		172.85 ± 33.59	177.93 ± 31.43	0.454
Diastolic blood pressure (mmHg)		87.91 ± 19.15	94.59 ± 17.22	0.078
Coronary artery disease		1 (2.9)	6 (8.8)	0.419
Diabetes mellitus		4 (11.8)	10 (14.7)	0.919
Chronic obstructive pulmonary disease		2 (5.9)	8 (11.8)	0.556
Left ventricular ejection fraction (%)		61.5 (57.0, 63.0)	63 (58.5, 66.0)	0.071
Left ventricular end-diastolic diameter (LVEDD) (mm)		46.5 (43.0, 49.0)	46.0 (42.5, 49.0)	0.533
Ascending aorta diameter (mm)		38.5 (36.3, 40.7)	39.0 (37.4, 41.6)	0.233
Aortic sinus diameter (mm)		36.5 (35.0, 42.8)	36.5 (34.3, 40.7)	0.650
Prior vascular surgery		2 (5.9)	1 (1.5)	0.257
Myocardial infarction		0 (0)	4 (5.9)	0.298
Cerebral infarction		2 (5.9)	4 (5.9)	1
Intestinal ischemia		0 (0.0)	7 (10.3)	0.092
Hepatic dysfunction		6 (17.6)	12 (17.6)	1
Renal dysfunction		6 (17.6)	18 (26.5)	0.322
Lower limb ischemia		0 (0.0)	1 (1.5)	1.000
Paraplegia		0 (0.0)	2 (2.9)	0.551
Location of the primary entry tear				
	Zone 2	1 (2.9)	2 (2.9)	0.837
	Zone 3	23 (67.6)	49 (72.1)	
	Zone 4	10 (29.4)	17 (25)	
Extent of dissection				
	DeBakey type Ⅲa	9 (26.5)	18 (26.5)	1
	DeBakey type Ⅲb	25 (73.5)	50 (73.5)	

Data are presented as n (%), mean ± SD, or median (Q1, Q3).

Statistical analyses were performed using SPSS version 26.0 (IBM-SPSS Statistics, Chicago, IL, USA). All statistical tests were two-sided, and a *p*-value < 0.05 was considered statistically significant. The normality of the distribution of continuous variables was first assessed using the Shapiro-Wilk test. Normally distributed variables were expressed as mean ± standard deviation (SD) and compared between groups using the independent samples *t*-test. Non-normally distributed variables were expressed as median (interquartile range) [M (Q1, Q3)] and compared between groups using the Mann-Whitney U test. Categorical variables were expressed as frequency (percentage) [n (%)] and compared between groups using the Chi-square test or Fisher’s exact test (when expected frequencies were <5). For multinomial categorical variables, such as postoperative branch artery perfusion patterns (true lumen/false lumen/mixed perfusion), if the overall test was significant, post-hoc pairwise comparisons with Bonferroni correction (adjusted α = 0.0167) were conducted to identify the source of the differences (see **Supplementary Post-hoc Analysis**). All statistical tests were two-sided, and a *p*-value < 0.05 was considered statistically significant. Post-hoc pairwise comparisons were conducted using Fisher’s exact test with Bonferroni correction (adjusted α = 0.0167). In addition, a post hoc power analysis was performed for the incidence of postoperative new entry tears based on the observed proportions in both groups, using a two-sided α level of 0.05.

## 3. Results

### 3.1 Baseline Patient Characteristics

A total of 102 patients were enrolled in this study, comprising 34 patients in the experimental group and 68 patients in the control group. The patient selection flowchart is presented in Fig. 2. There were no statistically significant differences between the two groups in baseline characteristics, including gender, age, time from symptom onset, smoking history, blood pressure, comorbidities (coronary artery disease, diabetes, chronic obstructive pulmonary disease, etc.), cardiac function, and aortic morphological parameters (all *p* > 0.05). The distribution of primary entry tear location and the extent of dissection were also well balanced between the two groups, indicating that the groups were comparable (Table 1).

### 3.2 Aortic True Lumen Remodeling

At the three-month postoperative follow-up, the true lumen area ratio at multiple anatomical planes was significantly better in the experimental group compared to the control group. Representative imaging findings of this remodeling are shown in Fig. 3. At the left subclavian artery plane, the median true lumen area ratio increased to 0.60 (0.41, 0.63) in the experimental group, which was significantly higher than the 0.41 (0.25, 0.54) observed in the control group (*p* = 0.002). At the pulmonary artery plane, the ratio significantly improved to 0.65 (0.54, 0.71) in the experimental group versus 0.44 (0.38, 0.53) in the control group (*p* < 0.001). Similarly, at the diaphragm plane, the ratio increased to 0.61 (0.50, 0.76) in the experimental group, significantly surpassing the 0.44 (0.37, 0.51) in the control group (*p* < 0.001). No statistically significant differences in the postoperative true lumen area ratio were found between the two groups at the renal artery plane (*p* = 0.180) or the iliac artery plane (*p* = 0.977) (Table 2).

**Table 2.** **True lumen area ratio before and after the procedure**.

Anatomical plane	Preoperative			Postoperative (3-month)		
	Experimental group (n = 34)	Control group (n = 68)	*p*-value	Experimental group (n = 34)	Control group (n = 68)	*p*-value
Left Subclavian Artery	0.39 (0.31, 0.46)	0.40 (0.25, 0.52)	0.451	0.60 (0.41, 0.63)	0.41 (0.25, 0.54)	0.002
Pulmonary Artery	0.45 ± 0.10	0.45 ± 0.11	0.814	0.65 (0.54, 0.71)	0.44 (0.38, 0.53)	<0.001
Diaphragm	0.39 ± 0.07	0.43 ± 0.10	0.062	0.61 (0.50, 0.76)	0.44 (0.37, 0.51)	<0.001
Renal Artery	0.51 (0.38, 0.58)	0.51 (0.45, 0.67)	0.142	0.54 (0.48, 0.83)	0.51 (0.40, 0.67)	0.180
Iliac Artery	0.64 (0.56, 0.84)	0.58 (0.50, 0.87)	0.261	0.84 (0.41, 1.00)	0.57 (0.50, 0.86)	0.977

Data are presented as n (%), mean ± SD, or median (Q1, Q3).

### 3.3 Improvement in Branch Artery Perfusion

Postoperatively, the experimental group demonstrated superior recovery of true lumen perfusion in key branch arteries. For the celiac trunk, the true lumen proportion increased from 79.4% (27/34) to 94.1% (32/34) in the experimental group, and from 69.1% (47/68) to 73.5% (50/68) in the control group, with a significant overall distribution difference between the groups (*p *= 0.027). Post-hoc analysis (see **Supplementary Material**) indicated that the difference primarily manifested as a significantly higher success rate of true lumen perfusion in the experimental group (97.0%) compared to the control group, where a higher proportion of mixed perfusion (23.1%) was observed (*p *= 0.011).

For the left renal artery, a significant overall difference in postoperative perfusion patterns was also found between the two groups (*p *= 0.017). Four cases (12.5%) in the experimental group exhibited mixed perfusion, whereas none (0%) in the control group presented with this pattern (*p *= 0.012). No significant differences were observed in the postoperative perfusion patterns of the superior mesenteric artery, right renal artery, or bilateral iliac arteries between the two groups (all *p* > 0.05) (Table 3).

**Table 3.** **Branch artery perfusion patterns**.

Artery	Perfusion type	Preoperative			Perfusion type	Postoperative (3-month)		
		Experimental group (n = 34)	Control group (n = 68)	*p*-value		Experimental group (n = 34)	Control group (n = 68)	*p*-value
Celiac trunk	True Lumen	27 (79.4)	47 (69.1)	0.547	True Lumen	32 (94.1)	50 (73.5)	0.027
	False Lumen	2 (5.9)	6 (8.8)		False Lumen	1 (2.9)	3 (4.4)	
	Mixed	5 (14.7)	15 (22.1)		Mixed	1 (2.9)	15 (22.1)	
Superior Mesenteric Artery	True Lumen	30 (88.2)	57 (83.8)	0.898	True Lumen	32 (94.1)	63 (92.6)	1.000
	False Lumen	1 (2.9)	3 (4.4)		False Lumen	0 (0.0)	2 (2.9)	
	Mixed	3 (8.8)	8 (11.8)		Mixed	2 (5.9)	3 (4.4)	
Left Renal Artery	True Lumen	22 (64.7)	56 (82.4)	0.119	True Lumen	28 (82.4)	62 (91.2)	0.017
	False Lumen	10 (29.4)	10 (14.7)		False Lumen	2 (5.9)	6 (8.8)	
	Mixed	2 (5.9)	2 (2.9)		Mixed	4 (11.8)	0 (0.0)	
Right Renal Artery	True Lumen	28 (82.4)	54 (79.4)	0.635	True Lumen	28 (82.4)	55 (80.9)	0.790
	False Lumen	2 (5.9)	8 (11.8)		False Lumen	2 (5.9)	7 (10.3)	
	Mixed	4 (11.8)	6 (8.8)		Mixed	4 (11.8)	6 (8.8)	
Left Iliac Artery	True Lumen	28 (82.4)	51 (75.0)	0.402	True Lumen	28 (82.4)	58 (85.3)	0.700
	False Lumen	-	-		False Lumen	-	-	
	Mixed	6 (17.6)	17 (25.0)		Mixed	6 (17.6)	10 (14.7)	
Right Iliac Artery	True Lumen	24 (70.6)	48 (70.6)	0.789	True Lumen	24 (70.6)	50 (73.5)	0.192
	False Lumen	2 (5.9)	2 (2.9)		False Lumen	2 (5.9)	0 (0.0)	
	Mixed	8 (23.5)	18 (26.5)		Mixed	8 (23.5)	18 (26.5)	

Data are presented as n (%).

### 3.4 Procedure-Related Complications

No statistically significant differences were observed in the overall incidence of complications between the two groups. The incidence of postoperative new entry tears was 23.5% (8/34) in the experimental group, lower than the 41.2% (28/68) in the control group, although this difference did not reach statistical significance (*p* = 0.079). A post hoc power analysis based on the observed incidence of postoperative new entry tears (23.5% vs. 41.2%) with sample sizes of 34 and 68 patients demonstrated a statistical power of 44% at a two-sided α level of 0.05. The rates of other complications, including intestinal ischemia, hepatic dysfunction, renal dysfunction, and lower limb ischemia, were low in both groups and showed no significant differences (all *p* > 0.05) (Table 4).

**Table 4.** **Comparison of procedure-related and postoperative complications**.

Variable	Experimental group (n = 34)	Control group (n = 68)	*p*-value
Postoperative new entry tears	8 (23.5)	28 (41.2)	0.079
Intestinal ischemia	0 (0.0)	2 (2.9)	0.551
Hepatic dysfunction	6 (17.6)	16 (23.5)	0.496
Renal dysfunction	2 (5.9)	8 (11.8)	0.556
Lower limb ischemia	1 (2.9)	1 (1.5)	1.000

Data are presented as n (%).

## 4. Discussion

This study comparing the efficacy of an integrated stent-graft system versus a conventional covered stent-graft alone for TBAD yielded several key findings. Compared to the control group, the experimental group demonstrated at three months postoperatively: (1) superior aortic true lumen remodeling, particularly at the left subclavian artery, pulmonary artery, and diaphragmatic planes; (2) better restoration of true lumen perfusion to critical branch arteries (celiac trunk and left renal artery); (3) a trend towards a lower incidence of postoperative new entry tears, without a significant increase in other complications. Collectively, these results suggest that the integrated stent-graft system may offer advantages in promoting aortic repair and maintaining end-organ perfusion, although causal inferences cannot be drawn due to the retrospective design.

### 4.1 Association Between Integrated Stent-Graft Use and Enhanced Aortic True Lumen Remodeling

In this study, the experimental group demonstrated significantly higher true lumen area ratios in the proximal and mid-aortic segments (left subclavian artery, pulmonary artery, and diaphragmatic planes) compared to the control group, which aligns with our initial hypothesis. The underlying mechanisms may involve several aspects.

#### 4.1.1 Sustained Radial Force of the Bare Stent and its Potential Clinical Benefits

A standard covered stent typically terminates distal to the primary entry tear, offering limited support to the uncovered true lumen beyond. The integrated stent-graft system in this study incorporates a 6 cm bare stent segment distal to the covered portion. This design provides sustained radial force to the aortic wall in the distal dissection segment, effectively countering passive compression from the false lumen and elastic recoil of the aortic wall, thereby establishing a stable “scaffold” for the expansion and maintenance of the true lumen. Studies confirm that this strategy of combining a proximal covered stent with a distal bare stent, compared to conventional TEVAR in treating complex TBAD, can significantly reduce postoperative complications and re-intervention rates, and more effectively promote aortic remodeling [16]. Specifically, implantation of the bare stent not only contributes to increasing the rate of complete false lumen thrombosis at the abdominal level but also reduces postoperative adverse events [17]. Furthermore, this supportive force, while maintaining true lumen patency, may play a role in preventing SINE tears, with related techniques (e.g., PETTICOAT) demonstrating significant advantages in mid-term follow-up.

#### 4.1.2 Promotion of False Lumen Thrombosis and Remodeling

Following the sealing of the primary proximal entry tear by the covered stent, blood flow within the false lumen becomes sluggish. The implantation of the distal bare stent, while expanding the true lumen, further alters the hemodynamics within the false lumen by reducing retrograde flow from distal re-entries. This promotes thrombosis and subsequent shrinkage of the false lumen, particularly in its mid-portion. This mechanism is supported by prior studies, which confirm that the combined strategy of a proximal covered stent and distal bare stent, compared to conventional TEVAR, can significantly increase the rate of complete false lumen thrombosis (including at the abdominal level) and effectively reduce the rates of postoperative re-intervention and SINE [18]. Furthermore, techniques such as PETTICOAT, which stabilize the distal intimal flap while promoting true lumen expansion and maintaining branch perfusion, have been shown to achieve more extensive aortic remodeling, especially in the abdominal aortic region [18,19]. The significant improvement in the true lumen area ratio at the diaphragmatic plane observed in our study is a direct manifestation of these hemodynamic improvements and the effect of false lumen thrombosis.

#### 4.1.3 Smooth Flow Transition and Improved Hemodynamics

The integrated design avoids the potential “step-off phenomenon” associated with the implantation of two separate stents within the diseased aorta, creating a smooth transition from the covered segment to the native aorta. This structural continuity helps reduce local flow disturbances, such as vortices and turbulent flow, thereby decreasing persistent shear stress on the aortic wall. This creates a more favorable hemodynamic environment for endothelial healing and long-term stability [20,21]. Studies indicate that designs promoting laminar flow and minimizing flow disturbance can effectively reduce the risk of stent migration or malapposition caused by flow impact [22], while a more stable flow pattern itself contributes to arterial wall repair [23]. Therefore, the integrated stent-graft system may enhance mechanical integrity through its physical connection and could potentially contribute to improved long-term patient outcomes via the smooth hemodynamic transition it provides.

### 4.2 Mechanisms and Clinical Significance of Improved Branch Artery Perfusion

Restoration of branch artery perfusion is a core objective in the treatment of TBAD. This study found that the experimental group was superior to the control group in restoring perfusion to the celiac trunk and the left renal artery.

#### 4.2.1 Celiac Trunk Perfusion: True Lumen vs. Mixed Perfusion

Postoperatively, the celiac trunk in the experimental group was almost exclusively perfused by the true lumen (97.0%), whereas a considerable proportion in the control group (23.1%) exhibited mixed perfusion. Mixed perfusion indicates the persistence of communications between the true and false lumens, with the false lumen continuing to supply the branch artery. This state is unstable, as either subsequent expansion or thrombosis of the false lumen can compromise the long-term blood supply to the branch. The integrated stent-graft system, via its distal bare stent segment, may more effectively compress the false lumen adjacent to the celiac trunk ostium, promoting thrombosis in this region [24,25], thereby establishing a purer and more stable true lumen perfusion pattern. Studies have shown that false lumen perfusion is associated with risks of end-organ (e.g., renal) dysfunction and atrophy [26,27]. Consequently, establishing stable true lumen perfusion is crucial for the long-term functional preservation of vital organs supplied by the celiac trunk, such as the liver, spleen, and stomach.

#### 4.2.2 Left Renal Artery Perfusion: The Unique Phenomenon of Mixed Perfusion

A noteworthy finding was the occurrence of mixed perfusion in the left renal artery in 4 cases (12.5%) within the experimental group, compared to none in the control group. This seemingly paradoxical result may actually reveal another mechanism of action of the bare stent. While forcefully expanding the true lumen, the implantation of the bare stent may induce a more extensive “fine-tuning” and active remodeling of the aortic intimal flap. Studies indicate that stent implantation can actively alter arterial architecture by increasing true lumen diameter, and its specific configuration can significantly influence the geometric angles and flow patterns of branch vessels [28]. In some cases, this proactive structural adjustment may transform an artery originally perfused exclusively by either the true or false lumen into a “mixed” state with flow from both lumens. This reflects a more aggressive and profound interventional effect of this technique on aortic anatomy. In contrast, the control group, due to its limited intervention scope, did not alter the original perfusion pattern. The Fisher’s exact test showing a significant difference in the overall distribution between the groups precisely indicates that the integrated stent-graft system introduces a novel pattern of anatomical remodeling, distinct from conventional therapy. Although the long-term clinical impact of this mixed perfusion requires further follow-up, its presence serves as a marker of the stent’s active local anatomical remodeling, carrying significant biomechanical and clinical implications.

#### 4.2.3 Clinical Relevance of Observed Changes in Perfusion

The improvement in true lumen perfusion observed in the experimental group is clinically relevant, as compromised branch vessel perfusion in acute aortic dissection may lead to renal dysfunction and visceral ischemia. Mixed or false lumen–dependent perfusion has been associated with unstable hemodynamics and a higher risk of malperfusion-related complications. Although the present study primarily focused on imaging-based assessment of perfusion patterns and aortic remodeling, enhanced true lumen perfusion may theoretically contribute to improved organ perfusion stability and reduced risk of late ischemic complications. However, we did not perform a dedicated analysis correlating perfusion patterns with functional outcomes such as renal function parameters or visceral ischemic events. Future studies incorporating longitudinal renal function testing and clinical symptom assessment are warranted to clarify the functional significance of perfusion pattern changes.

### 4.3 Trend in Postoperative New Entry Tears and Stent Mechanical Analysis

The incidence of postoperative new entry tears was lower in the experimental group than in the control group (23.5% vs. 41.2%); however, the difference did not reach statistical significance (*p* = 0.079). SINE is a serious and common complication following TEVAR, and its occurrence is closely associated with localized stress concentration on the aortic wall caused by the rigid edge of the stent-graft end [29].

The terminal end of a traditional covered stent, due to its abrupt rigid termination, is prone to creating peak stress points under the forces of blood flow and vessel pulsation, thereby potentially inducing new tears. In contrast, the integrated stent-graft system used in this study incorporates a flexible bare stent segment distal to the covered portion. This design creates a gradual, extended mechanical transition that disperses stress over a longer vascular segment, effectively reducing the risk of terminal stress concentration [30]. Furthermore, the support provided by the bare stent to the true lumen improves apposition to the aortic wall, reducing the “bird-beak” phenomenon of the stent and thereby enhancing the overall stability of the system.

However, given the limited statistical power (post hoc power of 44% for this outcome) and the short follow-up period, this result should be interpreted with caution. The observed trend is hypothesis-generating and requires validation in larger, adequately powered studies with long-term follow-up before any definitive conclusions can be drawn regarding the device’s potential to reduce SINE.

### 4.4 Limitations

First, several limitations related to sample size should be acknowledged. As this exploratory retrospective study evaluated a novel integrated stent-graft system, no formal pre-study sample size calculation was performed due to the lack of reliable preliminary effect size data (see section 2.8). The relatively small sample size, particularly in the experimental group (n = 34), may have limited statistical power to detect moderate differences. A post hoc power analysis based on the observed difference in postoperative new entry tear incidence (23.5% vs. 41.2%) showed a statistical power of 44% at a two-sided α level of 0.05, indicating a considerable risk of type II error. Therefore, the non-significant result (*p* = 0.079) should be interpreted cautiously and regarded as exploratory and hypothesis-generating, requiring confirmation in larger prospective studies.

Second, the follow-up period of this study was limited to 3 months. While this allows assessment of early aortic remodeling and short-term device-related outcomes, it is insufficient to capture clinically important long-term events in TEVAR for TBAD, including: (a) distal aortic remodeling progression or regression; (b) aneurysmal degeneration of the false lumen; (c) late distal stent-induced new entry (SINE); (d) reintervention rates; and (e) long-term survival. Although early imaging changes may suggest potential benefits, they do not necessarily translate into durable clinical benefit. Therefore, the long-term durability and stability of aortic repair cannot be established from the current data, and extended follow-up of 1–2 years or longer is needed. Ongoing follow-up of this cohort is underway to address these important outcomes.

In addition, an upper age limit of 75 years was applied to reduce confounding from age-related comorbidities and enhance internal validity in this preliminary evaluation. A conservative selection strategy was also adopted during early implementation of the device. This restriction may limit generalizability, and the benefit of this approach in patients >75 years requires further validation in larger multicenter studies. Finally, perfusion patterns were not systematically correlated with organ-specific functional outcomes, limiting full interpretation of the clinical impact of the imaging findings.

## 5. Conclusions

In this exploratory study of patients with TBAD, the integrated stent-graft system was associated with more favorable early aortic remodeling and improved true lumen perfusion to critical branch arteries compared to a conventional covered stent-graft alone. Specifically, the integrated stent-graft system demonstrated significantly higher true lumen area ratios in the proximal and mid-aortic segments and a higher rate of true lumen perfusion for the celiac trunk, along with a trend toward a lower incidence of new entry tears. However, given the short follow-up duration, relatively small sample size—particularly in the experimental group—and the exploratory nature of this study, these findings should be considered preliminary and hypothesis-generating. The early imaging changes observed may not necessarily translate into long-term clinical benefits such as reduced aneurysmal degeneration, reintervention, or improved survival. The statistical significance observed for some outcomes does not preclude the possibility of type II error for other clinically meaningful differences, and the robustness of these estimates requires confirmation in larger, adequately powered studies. Nevertheless, through its unique mechanical design, this integrated stent-graft system d a promising therapeutic strategy that warrants further investigation.

## Data Availability

The raw data of this article, which support the conclusions, will be made available by the authors, without undue reservation.
