# Supplementary material for: Comparative Efficacy of Integrated Stent-Graft versus Conventional Covered Stent-Graft Alone in Type B Aortic Dissection: A Retrospective Study Focusing on Aortic Remodeling and Branch Perfusion
Source: Rev Cardiovasc Med. 2026 Jun 24;27(6):49740. doi: 10.31083/RCM49740 (PMC13339603; doi:10.31083/RCM49740)
Supplement: Supplementary file 1 [file 2153-8174-27-6-49740-s1.zip › Supplementary Material.docx]

**Supplementary Material: Post-hoc Analysis**

**Supplementary Table S1. Post-hoc pairwise comparison of celiac trunk perfusion patterns (True lumen vs. False lumen)**

| Artery | Perfusion Type | Experimental Group (n=33) | Control Group (n=53) | *p*-value |
| --- | --- | --- | --- | --- |
| Ce-liac trunk | True Lumen | 32(97.0) | 50(94.3) | 1.000 |
|  | False Lumen | 1(3.0) | 3(5.7) |  |

Data are presented as n (%).

**Supplementary Table S2. Post-hoc pairwise comparison of celiac trunk perfusion patterns (True lumen vs. Mixed)**

| Artery | Perfusion Type | Experimental Group (n=33) | Control Group (n=65) | *p*-value |
| --- | --- | --- | --- | --- |
| Ce-liac trunk | True Lumen | 32(97.0) | 50(76.9) | 0.011 |
|  | Mixed | 1(3.0) | 15(23.1) |  |

Data are presented as n (%).

**Supplementary Table S3. Post-hoc pairwise comparison of celiac trunk perfusion patterns (False lumen vs. Mixed)**

| Artery | Perfusion Type | Experimental Group (n=2) | Control Group (n=18) | *p*-value |
| --- | --- | --- | --- | --- |
| Ce-liac trunk | False Lumen | 1(50.0) | 3(16.7) | 0.368 |
|  | Mixed | 1(50.0) | 15(83.3) |  |

Data are presented as n (%).

The overall analysis of postoperative Ce-liac trunk perfusion patterns revealed a significant intergroup difference (p = 0.027). To further clarify the source of this difference, Bonferroni-corrected post-hoc pairwise comparisons were performed (corrected α=0.0167). The results indicated a significant difference between the experimental and control groups in the distribution of true lumen perfusion versus mixed perfusion (p = 0.011 < 0.0167). Specifically, the experimental group demonstrated a higher success rate of true lumen perfusion (97.0%), whereas the control group showed a higher proportion of mixed perfusion (23.1%). Comparisons between other perfusion type pairs (true lumen vs. false lumen; false lumen vs. mixed) showed no statistical significance.

**Supplementary Table S4. Post-hoc pairwise comparison of Left Renal Artery perfusion patterns (True lumen vs. False lumen)**

| Artery | Perfusion Type | Experimental Group (n=30) | Control Group (n=68) | *p*-value |
| --- | --- | --- | --- | --- |
| Left Renal Artery | True Lumen | 28(93.3) | 62(91.2) | 1.000 |
|  | False Lumen | 2(6.7) | 6(8.8) |  |

Data are presented as n (%).

**Supplementary Table S5. Post-hoc pairwise comparison of Left Renal Artery perfusion patterns (True lumen vs. Mixed)**

| Artery | Perfusion Type | Experimental Group (n=32) | Control Group (n=62) | *p*-value |
| --- | --- | --- | --- | --- |
| Left Renal Artery | True Lumen | 28(87.5) | 62(100.0) | 0.012 |
|  | Mixed | 4(12.5) | 0(0.0) |  |

Data are presented as n (%).

**Supplementary Table S6. Post-hoc pairwise comparison of Left Renal Artery perfusion patterns (False lumen vs. Mixed)**

| Artery | Perfusion Type | Experimental Group (n=6) | Control Group (n=6) | *p*-value |
| --- | --- | --- | --- | --- |
| Left Renal Artery | False Lumen | 2(33.3) | 6(100.0) | 0.061 |
|  | Mixed | 4(66.7) | 0(0.0) |  |

Data are presented as n (%).

The significant overall difference in postoperative left renal artery perfusion patterns was primarily attributable to the distribution of mixed perfusion. Specifically, 12.5% of patients in the experimental group exhibited mixed perfusion, whereas none in the control group demonstrated this pattern after Bonferroni correction.

No significant differences were observed in isolated true lumen or false lumen perfusion patterns. Therefore, the observed intergroup difference was driven mainly by variation in mixed perfusion distribution rather than by an increase in true lumen perfusion alone.

Given the small sample size in some subgroups, these findings should be interpreted cautiously.
